# Viral hepatitis as an occupational disease in Poland

**Published:** 2011-07-01

**Authors:** Bartosz Bilski

**Affiliations:** 1 Department of Preventive Medicine, University of Medical Sciences, Poznan, Poland

**Keywords:** Hepatitis, Occupational disease, Epidemiology

## Abstract

**Background:**

In medical terms, occupational diseases are defined as health disorders specifically associated with the working environment of people and their occupational activity. From the medical and legal perspectives, the vast majority of European countries consider particular diseases to be of occupational origin if they are mentioned in the current list of occupational diseases and caused by exposure to factors in the working environment that are harmful to health.

**Objectives:**

The aim of this study was to analyze the occurrence of cases of viral hepatitis certified as an occupational disease in Poland during 1979-2009. This article presents the medical, economic, and legal aspects of the epidemiology of hepatitis as an occupational disease in Poland.

**Materials and Methods:**

Publically available statistical data on certified occupational diseases in Poland and data contained in individual "occupational disease diagnosis cards" (based on data used in Poland statistical form), regarding certified cases of hepatitis among health care professionals, which were collected by the Department of Occupational Hygiene of the Polish Public Health Service, were analyzed in this study.

**Results:**

In Poland, the highest number of cases of hepatitis certified as an occupational disease was observed in 1987. A gradual reduction in the number of cases of hepatitis as an occupational disease has been noted since then. Currently, hepatitis C as an occupational disease is certified more frequently than hepatitis B. In Poland, the number of women with hepatitis certified as an occupational disease is higher than that of men. However, among health care professionals, particularly nurses, this difference is insignificant because women outnumber the men. The existence of such a situation is due to the significant quantitative predominance of women over men among medical personnel, especially among nurses.

**Conclusions:**

Immunization of health care professionals against the hepatitis B virus (HBV), introduced in Poland in 1988, was an important factor involved in reducing the number of cases of occupational viral hepatitis. Socioeconomic and financial factors affected the epidemiological data on cases of hepatitis certified as an occupational disease in Poland. An additional problem associated with the diagnosis of occupational diseases is the lack of obligatory testing for anti-hepatitis C virus (HCV) and anti-hepatitis B surface antigen (HBsAg) antibodies and examinations to ensure the efficacy of HBV vaccination among medical staff before and during employment.

## 1. Background

In medical terms, occupational diseases are defined as health disorders specifically associated with the working environment of people and their occupational activity. From medical and legal perspectives, the vast majority of European countries consider particular diseases to be of occupational origin if they are mentioned in the current list of occupational diseases and are caused by exposure to factors in the working environment that are harmful to health. The European List of Occupational Diseases was prepared in 1990 and has been updated since then. The list is a variant of the European Commission's (EC) recommendation for European Union (EU) Member States. In Poland, the current list of occupational diseases includes 26 points, denoting groups of diseases and particular types of occupational diseases, and its scope is similar to that of the EC recommendations. As defined in Polish law, "Occupational diseases are recognized as such if included in the list of occupational diseases and considered to be obviously or most probably caused by exposure to deleterious factors in the working environment, or associated with occupational activities." In Poland, the procedure involved in the identification of occupational diseases is composed of 3 elements: suspicion of an occupational disease, its diagnosis by a physician possessing appropriate qualifications and employed by an authorized institution, and its certification by administrative proceedings carried out by the Public Health Service (in addition to the opportunity of recourse to law).

On the basis of the certification of an occupational disease, the affected employee is entitled to receive specific financial benefits. Therefore, official data on the number of cases of certified occupational diseases are analyzed not only from a medical perspective (the actual occurrence of the disease and access to suitable diagnostic services and treatment), as well as from a socioeconomic perspective. An individual's awareness of the disease and the labor market situation are also considered. These guidelines predominantly apply to diseases that cause permanent or chronic health impairment. Furthermore, in some diseases, access of the exposed individuals to suitable high-quality medical diagnosis may be of significance.

The occupational background of infectious diseases in Poland was considered in 1928. Ascariasis, glanders, tuberculosis, anthrax, tetanus, and diseases posing a risk for medical personnel were included in the group of occupational diseases. Currently, the vast majority of certified infectious occupational diseases in Poland are cases of hepatitis and tuberculosis among health care professionals and borreliosis among forest service workers [1][2][3]. According to official epidemiological data, the most common certified occupational diseases in Poland are hepatitis B and C; hepatitis A and D are much less common. In case of hepatitis A, the low number is associated with the low incidence of the disease in the general population (only several dozen cases are officially registered annually), with the acute course of the disease, and with the common lack of any constant or long-term health damage following recovery. From the financial perspective, an employee in Poland is entitled to compensation not because of contracting an occupational disease, but because of the persistent or long-term health damage caused by the disease. In case of hepatitis D, rare diagnosis is associated with the relatively low incidence of hepatitis B + D co-infections in the general population and is a result of the compulsory vaccination of medical personnel against the hepatitis B virus (HBV). However, cases of hepatitis D virus (HDV) infection have been noted among health care professionals in Poland. These were individuals who had previously been HBV carriers and who had become additionally infected with HDV via professional exposure to infected blood.

Certification of viral hepatitis as an occupational disease is a difficult process. Because infection with hepatotropic viruses may occur under conditions other than professional exposure to those microbes, care must be taken while evaluating the patient's working conditions. Non-occupational factors increasing the risk of infection, i.e., surgeries, blood transfusions, frequent hospitalizations, and diagnostic procedures, are additional obstacles to the certification processes. From the viewpoint of medical certification, the sole fact of performing professional duties posing a risk of accidental disruption of skin continuity or exposure of the mucosa does not allow an unequivocal definition of the professional exposure of an employee to of hepatitis. However, documented confirmation of an event of exposure of an individual employee to potentially infectious material provides a basis for considering the existence of a causal relationship between professional duties and the diagnosed disease. Unfortunately, a significant proportion of these events has not been recorded in the past [4]. Doctors, more often than nurses, have disregarded such situations [4]. Currently, a majority of institutions have so called "needlestick notebooks" and "individual exposure cards." Needlestick or other exposure to potentially infectious material is, of course, treated as an accident at work; even though it does not result in serious consequences (according to the Polish definition, an accident at work is "a sudden event caused by an external cause associated with work, causing injury or death").

It should also be noted that detection of anti-hepatitis C virus (HCV) antibodies or diagnosis of HBs carriers in itself is not considered as an occupational disease. Only the diagnosis of an acute or chronic inflammatory condition in the hepatic tissue constitutes a basis for the recognition of hepatitis as an occupational disease. Difficulties associated with the lack of serological examination for HCV and HBV in employees before they start performing their professional duties in health care institutions in Poland have also been observed. Therefore, an infection detected after a long asymptomatic course of the disease may be wrongly diagnosed as an occupational disease. Routin examination of health care professionals for the presence of anti-HCV, anti-human immune deficiency virus HIV, and anti-HBsAg was introduced in Poland in 1996, but this was cancelled a year later (in contrast to many European countries). Currently, whether these examinations are performed or not is left to the discretion of a doctor specializing in occupational medicine. However, compulsory tests include estimation of bilirubin and alanine aminotransferase levels in blood serum (tests that may yield false negative results). Unfortunately, standard protocols for diagnosis of health care professionals infected with HBV and HCV, in Poland, still need to be improved[5][6]. Common examinations of the efficacy of vaccination against HBV are not performed in Poland, and this prevents the discovery of non-responders.

It may be suspected that the incidence of carriers may be higher in some medical personnel than in the general population (a similar situation has been observed in other countries). Previous studies have shown that the percentage of health care professionals in Poland who have anti-HCV antibodies in their blood fluctuates from below 1.3% to over 3.2% of the average values for the Polish society, which have been estimated to be approximately 1.5-2.0% [7][8]. The risk factors for the presence of anti-HCV antibodies in medical personnel in Poland include work in therapeutic dialysis units, performing duties characteristic for mid-level medical personnel, and past surgical procedures [8]. Notably, in many countries, the problem of HCV infection has been recognized as a problem of auxiliary personnel, employees of hemodialysis wards, and surgeons [8][9][10]. Poland is considered to have a low incidence of HBV infection [11]. Earlier estimates have suggested that about 1-2% of the general Polish population have HBsAg, and about 20% have the anti-hepatitis B core antigen (HBc) [12].

## 2. Objectives

The aim of this study was to analyze the occurrence of cases of viral hepatitis certified as an occupational disease in Poland during the years 1979-2009.

## 3. Materials and Methods

Publically available statistical data on certified occupational diseases in Poland and data contained in individual "occupational disease diagnosis cards" regarding certified cases of hepatitis among health care professionals, which were collected by the Departments of Occupational Hygiene of the Polish Public Health Service, were analyzed in this study [3][13][14][15]. "Occupational disease diagnosis cards" are only statistical standards used in Poland (these are not medically or legally defined standards of diagnosis occupational diseases; the general and current rules of diagnostics and certification of viral hepatitis as an occupational disease in Poland and in EU have been presented in the background).

The following data were analyzed: number of cases of viral hepatitis certified as an occupational disease during the years 1979-2009 (including gender and age data) and the number of cases in which the patient was a medical professional. The collected data did not include any personal information The employment data included professionally active doctors (including dentists), nurses, midwives, laboratory diagnosticians, paramedics, medical technicians, and medical rescue team members. For example, in 2008, there were 78,086 doctors; 12,765 dentists; 182,778 nurses; 21,808 midwifes; 9,008 lab diagnosticians; and 7,743 paramedics professionally active in Poland (the population of Poland is approximately 38.5 million). Data on the number of employees in health care institutions, published by the Polish Main Statistical Office in Warsaw, were used for estimating the incidence coefficients of hepatitis in medical professionals [16]. Additional epidemiological examples were obtained from Wielkopolska province, where the author's University is located. Wielkopolska province is the second largest (29,825 km(2)) province in Poland and it has the third highest population (3.3 million).

## 4. Results

**Figure 1:**
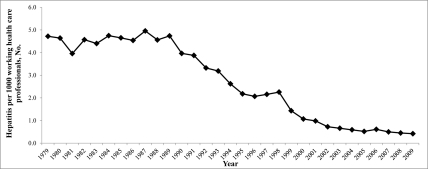
The number of hepatitis cases certified as an occupational disease per 1000 working health care professionals in the last 30 years in Poland

**Figure 2:**
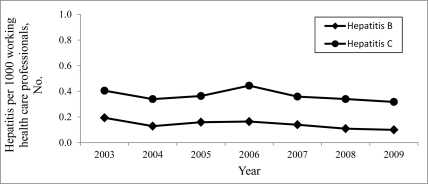
The number of hepatitis B and hepatitis C cases certified as occupational diseases per 1000 working health care professionals during the last years in Poland

**Figure 3:**
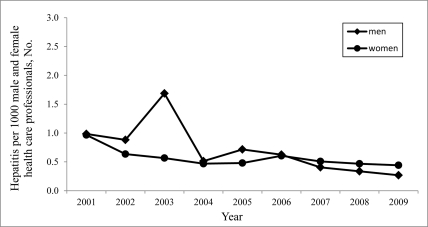
The number of hepatitis cases certified as an occupational disease among men and women  per 1000 working male and female health care professionals during the last years in Poland

In Poland, cases of hepatitis as an occupational disease were certified only among representatives of health care workers during 1979-2009. The highest number of cases of hepatitis certified as an occupational disease was noted in 1987 (Figure 1). Since then, the number of cases has gradually reduced (with a small inhibition in 1997-1998 and 2003). Currently, hepatitis C is certified as an occupational disease more frequently than hepatitis B was in the past years (Figure 2). In Poland, the number of women with hepatitis certified as an occupational disease is higher than that of men. However, among health care professionals, this difference is insignificant because women outnumber the men (Figure 3).

## 5. Discussion

The cases of hepatitis certified as an occupational disease in Poland during 1979-2009 occurred among medical professionals. This may be attributed to the fact that this group has the highest risk of hepatitis virus infection and probably, increased has awareness about medical certification. In Poland, the highest number of cases of viral hepatitis certified as an occupational disease was noted in 1987. Active immunization against HBV was introduced in Poland in 1988, and vaccination was first provided to medical personnel (for many years, this group had been administered booster vaccination every 5 years) (Figure 1). In 1993, the schedule of compulsory vaccination was expanded by immunization of patients before scheduled surgical procedures, common immunization of neonates in selected areas of the country, and in groups with high risk of HBV infection. Since 1997, all neonates and schoolchildren have been vaccinated against HBV, according to the following schedule: 0, 1, and 6 months (without booster doses). Intensification of vaccination in the 1990s caused a radical decrease in the number of new cases of hepatitis B in the general population. Before the introduction of general immunization against hepatitis B, Poland was an infamous regional record-breaker in the category of nosocomial HBV infections. This was because of the use of ineffective sterilization methods, lack of proper hygiene by medical personnel, and the utilization of multiple-use medical equipment.

Some inhibition of the reduction in the number of certified cases of viral hepatitis, both nationwide and in Wielkopolska province in 1997-1998 and 2003 (invasive specialties among men), was most probably caused by a reform in the health care system in Poland and the need for some financial security for health care professionals facing possible dismissal or because of a transformation of health care institutions (Figure 1 and Figure 3). From a medical perspective, cases of viral hepatitis certified as an occupational disease could be cases of hepatitis diagnosed many years ago, but employees took some steps towards the certification of their viral hepatitis as an occupational disease only in the face of some economic threat. The aim of certification was financial compensation for persistent or long-term damage to health. The situation in 1997-1998, 10 years after the introduction (1988) of the compulsory immunizations of medical personnel against HBV, was not a result of low efficacy, but of the fact that the period of time between medical diagnosis of the disease and its certification as an occupational disease may be quite long in Poland (however, in Poland, obligatory testing for anti-HBs antibodies after immunization to identify non-responders among health care workers has not been enforced).

The majority of cases of hepatitis certified as an occupational disease in Poland occurred among women (this trend has also been observed in Wielkopolska province [17]; approximately 83.3% of the cases in the country have been reported in women). This situation is associated with the significantly high number of medical personnel, particularly nurses, who are women. Occupational hepatitis occurs most often among nurses [17]. The differences are insignificant when we consider the number of male and female health care professionals in Poland (Figure 3).

The characteristic mean age of employees with hepatitis certified as an occupational disease is worth noting. In the past, hepatitis B has very often been certified among young nurses (aged 21-30 years), as described in a previous report [17]. This disturbing finding was mainly associated with the significant risk of blood-borne infections (common needlestick injuries and other forms of exposure to potentially infectious material), in the above-mentioned population easy transmission of HBV from patients to medical personnel, and lack of protective immunization against HBV at that time [18][19]. The mean age of employees with hepatitis certified as an occupational disease has increased lately. On average, the mean age is 46.0 ± 9.8 years for hepatitis B, and 45.4 ± 9.9 years for hepatitis C during 2001-2005 [1]. This epidemiological situation may be associated with the identification of hepatitis B certified as an occupational disease many years after medical diagnosis, or (hypothetically) with the lower immunity of older individuals to the infection, or the lower efficacy of immunization in older people. In the case of hepatitis C, the old age of employees in whom the disease is certified as an occupational disease may be associated with lower probability of HCV transmission (since viremia levels are lower hepatitis C than those in hepatitis B). Therefore, an employee has to be exposed to HCV more often to become infected with the virus. The deceptive course of HCV infection and the development of non-specific signs and symptoms may be additional factors that delay medical diagnosis, and consequently, the administrative certification of the disease as an occupational disease.

The problem associated with the diagnosis of occupational diseases in Poland is the lack of obligatory testing (for anti-HCV or anti-HBsAg antibodies) among the medical staff before and during employment. In Poland, the tests for anti-HBs antibody levels are not obligatory and are not widely performed. A doctor specializing in occupational medicine decides whether these examinations have to be performed or not (the decision also depends on the rules in a particular hospital). Preplacement medical examination and regular follow-up are necessary to document occupational hepatitis. For non-medical reasons (such as financial loss and risk of losing a job, etc.), an employee with earlier diagnosed infectious disease may start action aimed at the certification of this disease (i.e. hepatitis) as an occupational disease at any time. In conclusion, we state the following:

Immunization against HBV among health care workers (introduced in Poland in 1988) was an important factor affecting the reduction in the number of cases of viral hepatitis as an occupational disease in Poland. Socioeconomic and financial factors affected the epidemiological data on cases of hepatitis certified as an occupational disease in Poland. An additional problem associated with the diagnosis of occupational diseases in Poland is the lack of obligatory testing (for anti-HCV, anti-HBsAg antibodies, as well as examinations to estimate the efficacy of vaccinations against HBV) among medical staff before and during employment.
